# Strength and dynamic balance performance in soccer players in the United States: age, sex, and bilateral differences

**DOI:** 10.3389/fspor.2025.1510803

**Published:** 2025-01-22

**Authors:** Zhanxin Sha, Boyi Dai

**Affiliations:** ^1^ Exercise Science Department, Georgia Southwestern State University, Americus, GA, United States; ^2^ Department of Rehabilitation and Movement Science, The University of Vermont, Burlington, VT, United States; ^3^ Division of Kinesiology and Health, The University of Wyoming, Laramie, WY, United States

**Keywords:** asymmetry, push-up, countermovement jump, landing, reaching distance

## Abstract

Quantification of asymmetries between the two limbs is informative in assessing the risk of injury and performance deficits, but there is a paucity of studies investigating the effects of age and sex on bilateral asymmetry in young soccer players. This cross-sectional study aimed to investigate the effects of age and sex on strength and dynamic balance in 7- to 24-year-old soccer players in the United States. A total of 174 young soccer players participated in the study (Age 7–9 years: 26 females and 16 males; Age 10–12 years: 32 females and 31 males; Age 13–17 years: 17 females and 25 males; Age >18 years: 13 females and 14 males). Jump displacement, peak force, and asymmetry during countermovement jump with arm swing and landing, peak force and asymmetry during push-up, and normalized reaching distances for upper and lower extremity reaching tests were quantified. Preferred legs and arms were defined as the preferred kicking leg or throwing arm. As age increased, both preferred and non-preferred sides demonstrated decreased landing forces, increased jump displacement, and increased normalized peak forces during push-ups in both males and females (*p* < 0.05). Males showed greater jump displacement, normalized landing forces, and normalized peak forces in push-ups compared to females in several age groups (*p* < 0.05). No significant differences were observed for asymmetry variables between ages or sexes, and on average, most bilateral asymmetry variables were less than 5%. Age was associated with strength but not dynamic balance performance in healthy soccer players in the United States. Male and female players demonstrated similar changes, and bilateral asymmetries were on average small. Soccer players may need more dynamic balance training over time as they progress to higher levels of competition. Landing technique training may be implemented for young soccer players to decrease the high impact landing forces and landing related injury risk. Asymmetries and their relationships with injury risk should be evaluated on an individual basis, as their relationships with age and sex were weak. Future longitudinal and cohort studies are warranted to further elucidate the relationship among strength, dynamic balance, and injury risk in soccer players.

## 1 Introduction

Soccer is highly demanding from a physical perspective, as players need to perform various patterns of movement, such as repeated maximal or near maximal, multi-directional, unilateral, and asymmetric movements, as these play a significant role in the crucial moments of a match (1, 2). Moreover, the physical demands of soccer may differ significantly in relation to factors such as the players' age, competitive level, and the playing style of any given team (2). Over a long period, a high volume of training and competition might lead to differences in strength, power, and dynamic balance performance between limbs (1–3). Thus, quantification of bilateral asymmetry for the upper and lower extremities is critical to determine whether an individual may be at risk of injury or performance deficits (3–6).

Investigating bilateral limb asymmetries consists of comparisons between right and left, preferred and non-preferred, dominant and non-dominant sides or limbs in physical functions such as strength and dynamic balance (3, 4, 6–8). Strength is the ability of a specific group of muscles to produce maximum force to overcome an external load. Dynamic balance refers to the ability to maintain the center of gravity above the base during movement, even when the body moves outside the center of gravity during dynamic tasks (5, 8–11). Following strength and dynamic balance assessments, an athlete's profile could be established, and bilateral asymmetry information could be used as a reference to guide return-to-play decisions after injury to reduce the risk of reinjury or to optimize the training and rehabilitation programs (6, 7, 12). Commonly used parameters obtained from strength and dynamic balance tasks include peak force/torque, reaching distances, and bilateral asymmetry (7, 8, 13–15).

Age and sex likely affect the development of physical functions and might affect bilateral asymmetries in young soccer players. It has been shown that strength and jump height increase with age, and males generally perform better than females in vertical jump and push-up assessments (10, 16). However, there were inconsistent results regarding impact force and bilateral asymmetry of impact force between sexes during the landing phase (17). Only one study indicated that dynamic balance asymmetry was only slightly influenced by age in German youth soccer players (18). There is a paucity of investigation into the development of dynamic balance in soccer players in the United States. While previous studies have suggested that bilateral asymmetries are small in healthy collegiate athletes across a variety of sports (7), bilateral asymmetries might be more pronounced for lower extremity performance in soccer players who generally have a preferred kicking leg. However, there is a lack of studies quantifying bilateral asymmetries in strength and dynamic balance tasks in youth soccer players. Determining the prevalence of strength, dynamic balance, and bilateral asymmetry among different age groups would assist in creating individualized training plans and injury prevention strategies.

Therefore, the aim of the study was to investigate the effects of age and sex on strength and dynamic balance in 7- to 24-year-old soccer players in the United States. It was expected that soccer players would increase their strength and dynamic balance performance as they grow. Further, previous studies have shown that males generally perform better than females in strength performance, and bilateral asymmetries are generally small in healthy athletes. The first hypothesis was that strength and dynamic balance performance would significantly increase as age increased, while asymmetries would show minimal changes as a function of age. The second hypothesis was that males would show greater strength performance and non-significantly different dynamic balance performance and asymmetries compared to females. With this information, we can better understand the role of age and sex on the mechanisms of performance changes and injury risk factors. Consequently, the findings could contribute to the long-term goals of establishing age- and sex-specific injury risk screening, performance training, and injury prevention strategies.

## 2 Materials and methods

### 2.1 Participants

Based on a medium-to-large effect size of 0.8 between two age groups or two sex groups, a sample size of 26 was needed for each age or sex group to achieve a power of 0.8 at a type-I error level of 0.05. A total of 174 soccer players in four age groups participated in the study (7–9 years old: 26 females, 16 males; 10–12 years old: 32 females and 31 males; 13–17 years old: 17 females and 25 males; more than 18 years old: 13 females and 14 males; Table 1) participated in the study. Players were recruited from local youth soccer programs, collegiate clubs, and NCAA collegiate teams. All players regularly participated in organized soccer practice at least 1 time per week. Individuals were excluded if they possessed any musculoskeletal, cardiovascular, respiratory, neurologic, or other conditions that prevented them from participating in sporting activities. Participants between 7 and 13 years old agreed to a youth assent, and at least one of their parents signed a consent form. Participants between 14 and 18 years old and at least one of their parents both signed a consent form. Participants older than 18 years signed a consent form. The current study was approved by the University Institutional Review Board.

**Table 1:** Participant information (mean ± standard deviation).

		Age 7–9 years	Age 10–12 years	Age 13–17 years	Age >18 years
Age (years)	Male	8.4 ± 0.62	11.4 ± 0.84	15.1 ± 1.57	20.2 ± 1.81
	Female	8.7 ± 0.87	11.2 ± 0.82	15.2 ± 1.56	19.6 ± 0.84
Body height (cm)	Male	133.9 ± 5.2	148.8 ± 8.1	169.5 ± 9.7	178.80 ± 8.1
	Female	133.0 ± 6.6	148.2 ± 7.9	160.9 ± 7.4	169.0 ± 6.2
Body weight (*N*)	Male	280.1 ± 44.5	372.8 ± 71.1	606.5 ± 125.5	713.1 ± 80.1
	Female	277.1 ± 49.8	384.3 ± 68.3	532.1 ± 113.2	608.4 ± 75.6

### 2.2 Measures

#### 2.2.1 Participant descriptors

Participants' ages in years were calculated based on their self-reported dates of birth. Participants' height was measured using a stadiometer or a tape measure placed on a vertical wall. Participants' body weight was captured during the standing phase of the countermovement jump with arm swing (CMJ-AS). Right leg length was measured from the anterior superior iliac crest to the medial malleolus. Right arm length was measured from C7 to the tip of the longest finger. Only the right limb length was measured to be consistent with a previous study (7). Preferred legs and arms were determined by the participant's self-reported preferred kicking leg or throwing arm for a further distance, respectively.

#### 2.2.2 Strength assessment

CMJ-AS was used to assess lower extremity dynamic strength and landing performance. Two portable force platforms (Bertec Corporation, Columbus, OH, USA) were used for measurements at a sampling frequency of 1,000 Hz (7). The successful trial was to jump vertically for a maximal height and land back on the force platforms. No specific instruction regarding landing techniques was given to participants. A minimum of 1 practice trial and 3 official trials were performed with a minimum of 15 s rest between trials.

Push-up assessments were also performed using two force platforms (7). The participants started with each hand below the shoulder and on a force platform and feet on a wooden platform with the same height. Participants lowered their body and pushed as fast and as forcefully as possible. A minimum of 1 practice trial and 3 official trials were performed with a minimum of 15 s rest between trials.

#### 2.2.3 Dynamic balance assessment

A Y-balance apparatus (Move2Perform, Evansville, IN, USA) was used to measure reaching distances for the upper and lower extremity reaching tests. For the lower extremity reaching test, each participant stood on one leg behind the starting line and reached with the free leg in the anterior direction (7). The participant could not touch the ground until they returned to the starting position. A minimum of 1 practice trial and 3 official trials were performed for each leg.

For the upper extremity reaching test, the participant started in a push-up position with the testing arm on the center plate. The participant reached laterally with the free arm (7). The participants could not touch the ground until they returned to the starting position. The maximal reaching distance was recorded. A minimum of 1 practice trial and 3 official trials were performed for each arm.

### 2.3 Data process and reduction

Vertical ground reaction forces were filtered using a fourth-order, zero-phase-shift Butterworth filter at a low-pass cutoff frequency of 100 Hz. The peak forces during the jumping and landing phases of CMJ-AS and the pushing phase of the push-up were extracted for each limb and normalized to body weight. Jump displacement was calculated using the impulse-momentum theorem (7). For peak jumping, landing, and pushing forces, the average of 3 trials was used for analysis. For the reaching tests, the greatest reaching distance of 3 trials was used and normalized to the limb length for analysis. The asymmetry index was calculated as follows: (preferred side—non-preferred side)/(preferred side). As such, positive indices indicated greater numbers on the preferred side. Negative indices indicated greater numbers on the non-preferred side. The calculations were performed using subroutines developed in MATLAB (MathWorks, Inc., Natick, MA, USA).

### 2.4 Statistical analysis

Data were analyzed using the Statistical Program for the Social Sciences 14.0 for Windows (SPSS, Inc., Chicago, IL, USA). Participants were grouped into eight categories based on sex (male/female) and four age subgroups: 7–9 years old, 10–12 years old, 13–17 years old, and more than 18 years old. Dependent variables included upper body reaching distance, lower body reaching distance, asymmetries of upper and lower reaching distance scores, jump displacement, normalized peak jumping and landing forces from the CMJ-AS, asymmetry of CMJ-AS peak jumping and landing forces, normalized peak forces from push-up, and asymmetry of peak forces from push-up. An analysis of variance (ANOVA) test was used to compare variables between sexes and age groups. Whenever the ANOVA revealed a significant factor (*p* < 0.05), *post hoc* pairwise comparisons were performed. Cohen's d, calculated as (Mean 1–Mean 2)/Pooled Standard Deviation, was used to evaluate the effect sizes of pairwise comparisons. Effect sizes were considered small (Cohen's d ≤ 0.5), medium (0.5 < Cohen's d < 0.8), or large (Cohen's d ≥ 0.8) (19).

## 3 Results

Means and standard deviations are presented in Tables 2, 3, while *p*-values of pairwise comparisons and effect sizes are included in Tables 4–7. Age had a significant effect on preferred and non-preferred leg reaching performances (Table 2). More than 18 years age group demonstrated decreased reaching distances compared to 7–9 years and 10–12 years age groups (5%–11% decreases for males and females, Cohen's d = 0.60–0.74, Table 4). For arm reaching performance, sex had a significant effect on preferred arm reaching performance with males reaching greater distances compared to females (7% greater for the oldest age group, Cohen's d = 0.34, Table 5).

**Table 2:** Performance and asymmetries in upper and lower extremity reaching tests (mean ± standard deviation).

		Age 7–9 years	Age 10–12 years	Age 13–17 years	Age >18 years	*p*-values of ANOVAs		
						Sex	Age	Interaction
Preferred leg reaching distance (leg length ratio)	Male	0.77 ± 0.10 A	0.78 ± 0.08 A	0.76 ± 0.11 AB	0.74 ± 0.09 B	0.886	0.033	0.497
	Female	0.81 ± 0.12 A	0.79 ± 0.06 A	0.75 ± 0.06 AB	0.72 ± 0.06 B			
Non-preferred leg reaching distance (leg length ratio)	Male	0.79 ± 0.09 A	0.79 ± 0.09 A	0.77 ± 0.11 AB	0.76 ± 0.10 B	0.403	0.012	0.360
	Female	0.81 ± 0.09 A	0.79 ± 0.06 A	0.75 ± 0.07 AB	0.71 ± 0.04 B			
Asymmetry of Lower leg reaching (%)	Male	−0.03 ± 0.05	−0.01 ± 0.06	−0.02 ± 0.06	−0.02 ± 0.06	0.090	0.797	0.821
	Female	−0.01 ± 0.08	−0.01 ± 0.06	0.01 ± 0.04	0.00 ± 0.05			
Preferred arm reaching distance (arm length ratio)	Male	0.94 ± 0.12^a^	0.99 ± 0.08^a^	0.98 ± 0.12^a^	1.01 ± 0.11^a^	0.030	0.660	0.348
	Female	0.95 ± 0.07^a^	0.96 ± 0.10^a^	0.92 ± 0.08^a^	0.94 ± 0.10^a^			
Non- preferred arm reaching distance (arm length ratio)	Male	0.96 ± 0.12	0.99 ± 0.07	0.98 ± 0.12	1.01 ± 0.09	0.051	0.577	0.693
	Female	0.96 ± 0.08	0.97 ± 0.09	0.94 ± 0.06	0.96 ± 0.09			
Asymmetry of arm reaching (%)	Male	−0.02 ± 0.09	0.00 ± 0.06	0.00 ± 0.06	0.00 ± 0.06	0.591	0.953	0.664
	Female	0.00 ± 0.08	−0.01 ± 0.06	−0.02 ± 0.09	−0.03 ± 0.05			

^a^ Statistics significantly different between males and females. A, B, C, and D: significantly different among the groups. A is the greatest, B is the second greatest, C is the third greatest, and D is the least.

**Table 3:** Performance and asymmetries in countermovement jump with arm swing, landing, and push-up.

		Age 7–9 years	Age 10−12 years	Age 13–17 years	Age >18 years	*p*-values of ANOVAs		
						Sex	Age	Interaction
Jump displacement (cm)	Male	19.2 ± 3.5 D	24.0 ± 5.1 C^a^	30.5 ± 5.2 B^a^	38.8 ± 6.0 A^a^	<0.001	<0.001	<0.001
	Female	19.5 ± 3.0 C	21.5 ± 3.1 B^a^	25.1 ± 6.4 A^a^	27.7 ± 3.6 A^a^			
Peak jumping force from preferred leg (body weight)	Male	1.21 ± 0.14	1.23 ± 0.15	1.21 ± 0.14	1.23 ± 0.20	0.166	0.707	0.289
	Female	1.30 ± 0.21	1.22 ± 0.12	1.29 ± 0.22	1.21 ± 0.14			
Peak jumping force from non-preferred leg (body weight)	Male	1.16 ± 0.14^a^	1.21 ± 0.16^a^	1.19 ± 0.14^a^	1.23 ± 0.16^a^	0.046	0.994	0.250
	Female	1.28 ± 0.18^a^	1.23 ± 0.13^a^	1.27 ± 0.22^a^	1.21 ± 0.15^a^			
Asymmetry of peak jumping force (%)	Male	0.04 ± 0.10	0.01 ± 0.07	0.02 ± 0.06	0.00 ± 0.07	0.308	0.362	0.764
	Female	0.01 ± 0.09	−0.01 ± 0.07	0.02 ± 0.07	0.00 ± 0.04			
Peak landing force from preferred leg (body weight)	Male	4.60 ± 1.46 A^a^	3.70 ± 1.87 AB^a^	2.87 ± 0.82 B^a^	3.30 ± 0.50 B^a^	0.004	<0.001	0.196
	Female	3.62 ± 1.26 A^a^	3.14 ± 1.41 AB^a^	3.00 ± 1.05 B^a^	2.25 ± 0.63 B^a^			
Peak landing force from non-preferred leg (body weight)	Male	4.14 ± 1.90 A^a^	3.78 ± 1.80 AB^a^	2.71 ± 0.80 BC^a^	3.00 ± 0.75 C^a^	0.007	0.002	0.104
	Female	3.29 ± 1.28 A^a^	2.89 ± 1.17 AB^a^	3.03 ± 1.17 BC^a^	2.11 ± 0.60 C^a^			
Asymmetry of Peak landing force (%)	Male	0.11 ± 0.26	−0.03 ± 0.18	0.05 ± 0.14	0.08 ± 0.24	0.857	0.182	0.258
	Female	0.08 ± 0.17	0.06 ± 0.20	0.00 ± 0.25	0.05 ± 0.20			
Peak pushing force from preferred arm (body weight)	Male	0.47 ± 0.04 CD	0.48 ± 0.05 C	0.57 ± 0.08 AB^a^	0.61 ± 0.08 A^a^	<0.001	<0.001	<0.001
	Female	0.49 ± 0.06 AB	0.46 ± 0.05 B	0.49 ± 0.07 AB^a^	0.50 ± 0.07 A^a^			
Peak pushing force from non-preferred arm (body weight)	Male	0.46 ± 0.04 CD	0.47 ± 0.05 C	0.56 ± 0.09 AB^a^	0.62 ± 0.09 A^a^	<0.001	<0.001	<0.001
	Female	0.48 ± 0.08 AB	0.46 ± 0.06 B	0.47 ± 0.04 AB^a^	0.51 ± 0.08 A^a^			
Asymmetry of peak pushing (%)	Male	0.02 ± 0.09	0.02 ± 0.09	0.02 ± 0.06	0.00 ± 0.06	0.814	0.431	0.951
	Female	0.02 ± 0.08	0.01 ± 0.09	0.03 ± 0.05	−0.01 ± 0.04			

^a^ Statistics significantly different between males and females. A, B, C, and D: significantly different among the groups. A is the greatest, B is the second greatest, C is the third greatest, and D is the least.

**Table 4:** *P*-values of pairwise comparisons and Cohen's d among age groups for variables showing main age effects.

		7–9 years vs. 10 -12 years	7–9 years vs. 13 −17 years	7–9 years vs. > 18 years	10–12 years vs. 13–17 years	10–12 years vs. > 18 years	13–17 years vs. > 18 years
Preferred leg reaching distance	*p*-value	0.581	0.118	0.018	0.092	0.002	0.253
	Cohen's d	0.11	0.35	0.60	0.34	0.74	0.28
Non-preferred leg reaching distance	*p*-values	0.454	0.054	0.005	0.094	0.004	0.297
	Cohen's d	0.15	0.43	0.73	0.34	0.67	0.26
Peak landing force from preferred leg	*p*-values	0.069	<0.001	<0.001	0.087	0.064	0.523
	Cohen's d	0.37	0.90	1.00	0.35	0.43	0.16
Peak landing force from non-preferred leg	*p*-values	0.386	0.010	0.002	0.078	0.020	0.232
	Cohen's d	0.17	0.58	0.78	0.36	0.55	0.30

Male and female data were combined.

**Table 5:** *P*-values of pairwise comparisons and Cohen's d between males and females for variables showing main sex effects.

Preferred arm reaching distance	*p*-values	0.028
	Cohen's d	0.34
Peak jumping force from non-preferred leg	*p*-values	0.031
	Cohen's d	0.33
Peak landing force from preferred leg	*p*-values	0.036
	Cohen's d	0.32
Peak landing force from non-preferred leg	*p*-values	0.018
	Cohen's d	0.36

Different age groups were combined.

**Table 6:** *P*-values of pairwise comparisons and Cohen's d among age groups for variables showing interaction effects.

		7–9 years vs. 10–12 years	7–9 years vs. 13–17 years	7–9 years vs. >18 years	10–12 years vs. 13–17 years	10–12 years vs. >18 years	13–17 years vs. >18 years
Male							
Jump displacement	*p*-values	0.002	<0.001	<0.001	<0.001	<0.001	<0.001
	Cohen's d	1.03	2.43	4.07	1.25	2.74	1.53
Peak pushing force from preferred arm	*p*-values	0.852	<0.001	<0.001	<0.001	<0.001	0.122
	Cohen's d	0.06	1.45	2.17	1.43	2.15	0.56
Peak pushing force from non-preferred arm	*p*-values	0.681	<0.001	<0.001	<0.001	<0.001	0.091
	Cohen's d	0.13	1.28	2.17	1.27	2.21	0.62
Female							
Jump displacement	*p*-values	0.021	<0.001	<0.001	0.011	<0.001	0.199
	Cohen's d	0.63	1.21	2.55	0.81	1.90	0.48
Peak pushing force from preferred arm	*p*-values	0.070	0.795	0.537	0.219	0.034	0.471
	Cohen's d	0.49	0.08	0.21	0.38	0.73	0.27
Peak pushing force from non-preferred arm	*p*-values	0.174	0.626	0.366	0.496	0.013	0.170
	Cohen's d	0.37	0.16	0.31	0.21	0.86	0.53

Male and female data were separated.

**Table 7:** *P*-values of pairwise comparisons and Cohen's d between males and females for variables showing interaction effects.

		7–9 years	10–12 years	13–17 years	>18 years
Jump displacement	*p*-values	0.775	0.021	0.005	<0.001
	Cohen's d	0.09	0.60	0.94	2.24
Peak pushing force from preferred arm	*p*-values	0.334	0.295	0.001	0.002
	Cohen's d	0.31	0.27	1.11	1.39
Peak pushing force from non-preferred arm	*p*-values	0.331	0.488	0.003	0.003
	Cohen's d	0.31	0.18	1.04	1.32

Different age groups were separated.

An interaction effect was observed for countermovement jump displacement (Table 3), suggesting males and females demonstrated different changes as a function of age. The post-hoc comparison revealed that jump displacement generally increased as age increased (42% increases for females and 100% increases for males and Cohen's d = 2.55–4.07 from the youngest to the oldest age groups, Table 6), and males showed higher jump displacement in participants older than 10 years than females (30% higher and Cohen's d = 2.24 for the oldest age group, Table 7). Significant main age and sex effects were observed for normalized landing forces. 7–9 years age group demonstrated increased normalized landing forces for both preferred and non-preferred legs compared to 13–17 age and more than 18 years age groups (28% increases for males and 38% increases for females and Cohen's d = 0.78–1.00 between the youngest and the oldest age groups, Table 4), and males showed greater normalized landing forces than females (32% increases and Cohen's d = 0.32–0.36, Table 5).

Significant interaction effects were observed for normalized peak pushing forces for both preferred and non-preferred arms (Table 3). Normalized peak pushing forces were the greatest in more than 18 years age group (27% increases for males and 8% increases for females and Cohen's d = 0.73–2.21 compared to the second youngest group, Table 6), and males showed greater normalized peak pushing forces than females (18% greater and Cohen's d = 1.32–1.39 for the oldest age group, Table 7). No significant age or sex effects were observed for any strength or dynamic balance asymmetry variables. On average, bilateral asymmetries were all below 5%, except for landing force asymmetries, which were greater than 5% for certain age and sex groups.

## 4 Discussion

The findings supported the hypotheses that strength would significantly increase as age increased while bilateral asymmetries would show non-significant changes as a function of age, but the findings did not support that dynamic balance would significantly increase as a function of age. Meanwhile, the findings supported the hypothesis that males would show greater strength and non-significantly different dynamic balance and bilateral asymmetries than females.

### 4.1 Strength assessment

The current study was consistent with previous studies (20, 21) that older players showed better performance in vertical jump displacement in both males and females. There was no statistically significant difference in normalized peak jumping force between different age groups. Males showed higher jump displacements than females in participants older than 10 years, but the peak normalized jumping forces were non-significantly different between males and females. This may be due to the vertical jump being a complex multi-segment movement, and many factors contribute to vertical jump performance. For example, lean body mass, muscular strength, and coordination between extremities are all contributors to vertical jump performance (7, 22, 23). Better vertical jump performance may require higher force output throughout the jump (impulse), not merely one peak force value (7, 23). In addition, the critical time periods in the vertical jump also play an important role. Athletes with better jump performance produced more force at the key time intervals of 50 ms, 90 ms, and 250 ms (22, 23). Thus, the higher performing jumpers are more explosive athletes who can generate larger impulses during the propulsive phase of the vertical jump (23). That could partly explain why there was no difference in normalized peak force between age groups, but older groups had better vertical jump performance.

The current study observed the effects of age and sex on the peak magnitude of landing forces at both preferred and non-preferred legs. Younger individuals exhibit greater normalized peak landing forces from countermovement jumps. Generally, male soccer players had greater normalized peak landing forces compared to female soccer players, likely due to the higher jump displacement in males than females. A previous longitudinal study showed male elite soccer players improved landing kinematics from 13 years old to 17 years old (24). In the current study, the improvement of landing techniques to decrease impact forces may be related to neuromuscular changes occurring during growth, as well as to learning, training, and exposure to preventing landing-related injuries.

The result may provide additional evidence that young players may be more prone to landing injuries because of a lack of landing technique training and neuromuscular control of safe movement patterns (3, 24). Due to the high impact forces during landing, players under 14 years of age may be vulnerable to injury (3, 24–26). The results of this study increase our knowledge about the function of landing mechanics relative to age-related differences in strength and motor control strategies and have important implications for using early intervention to effectively teach motor skills in children and reduce the risk of injuries (e.g., motor skills that require timing, neuromuscular re-education, and bilateral symmetry, neurofeedback, unilateral, and bilateral training, etc.) (25, 27, 28).

Males generated greater normalized peak forces than females in the push-up assessment, which is consistent with previous studies (7, 16). Both intrinsic and extrinsic factors could explain the differences in performance between males and females. Factors include but are not limited to skeletal activation, neuromuscular control levels, training strategies, and motor control strategy (7, 10, 29). These factors are highly dynamic, trainable, and individual-based and can be improved with training and maturation (3, 10, 30). Age also has a significant positive effect on normalized peak force from both the preferred and non-preferred sides during push-ups. Soccer is classified as a contact/collision sport, and players need to protect the ball or fend off opponents, which requires upper-body strength and power. Thus, training and assessing upper-body strength are essential for soccer players.

Consistent with previous findings that normalized peak force bilateral asymmetry from push-ups and CMJ-AS were generally within 10% in the current study (7, 16, 31, 32). The current study included only soccer players ranging from 7 to 24 years old. There were no significant bilateral asymmetry differences across the different age groups. Soccer games demand mainly sprint actions, lateral movements, jumps, changes of direction, etc. Although a player may prefer the preferred leg for certain kicking motions, these motions alone did not appear to lead to bilateral asymmetries. As such, it should not be assumed that age alone will increase bilateral asymmetries in soccer players.

### 4.2 Dynamic balance

The current study measured anterior reach from lower YBT because the anterior reaching distance was found to be associated with injuries (33). An age effect was observed on both preferred and non-preferred legs in the reaching test. Our findings were partially consistent with Chimera et al. (34) in that sex differences were not likely to affect dynamic balance performance on the YBT in all age groups. In our study, the oldest groups showed decreased normalized leg reaching distances compared to the youngest group in both the male and female groups. The result from the current study supports that dynamic balance ability should not be assumed to increase as a function of age.

Compared to lower extremities YBT, there was a paucity of studies investigating upper extremities YBT (13, 35, 36). Assessment of the upper extremity is helpful for identifying the risk of running-related injuries (hip/thigh/knee injuries) due to instability and deficiencies in the core muscles. Therefore, periodic assessment of upper extremity reaching distance may help identify the core physical status of soccer players. A previous study found age effects on shoulder mobility and reaching distance in handball players (36). The current study did not find a similar trend but found that sex had a significant effect on arm reaching performance from YBT. Consistent with Ruffe et al. (35), there was no difference in reaching distance across age groups after normalizing the arm length from the current study. It may be related to sport-specific training and adaptations. Handball players involve more unilaterally throwing, passing, and shooting actions (36). Soccer players are likely to use their upper extremities to coordinate ball kicking, shield the ball, or fend off opponents during practice and competitions.

There is a paucity of studies investigating age and sex effects in bilateral asymmetry. The results were not consistent due to the testing method and age of participants in the previous studies. A previous study indicated that male collegiate athletes showed more asymmetry than their female counterparts (34). The current study did not find a difference in asymmetry between males and females across different age groups. One of the reasons is that the current study only recruited healthy soccer players; thus, most asymmetries were below 5%. That may also indirectly support the idea that healthy athletes should have asymmetrical scores lower than 10%. In addition, there are many factors related to dynamic balance performance. Butler et al. (37) indicated that dynamic balance could be relative to sport, competition level, training background, maturity status, and sex after comparing adolescent male soccer players in Rwanda and the US.

When interpreting normative asymmetries, it is important to consider that athletes' asymmetry performance might be more individual-based than sports-specific. A certain degree of lower asymmetry performance among collegiate athletes may not affect change of direction performance. In addition, studies have indicated that a task-specific nature of asymmetries and correlations has been observed in some tasks but not in others (6, 7). Thus, the same group of athletes may have different bilateral asymmetry performances based on the types of assessments. Different from a previous study (33), but consistent with Buoite Stella et al. (38), the current study did not find asymmetry in upper and lower YBT between males and females across four different age groups. The reason might be related to the background of participants from the studies. The previous study recruited participants with a history of injuries and non-injuries from different sports (33). The current study only recruited healthy soccer players from different age groups. Previous investigation indicated that asymmetry indices may vary depending on the individual differences and the task used to quantify limb balance as well as after testing different asymmetry index matrices such as change of direction, unilateral jump, and functional movement screen in youth basketball players (39, 40). Similarly, after comparing basketball and handball athletes, the study also found that the type of sport might influence dynamic balance as well (32). Even though the relationship between asymmetries and sports performance has not been consistent, with potential links to injury risk, the analysis of interlimb asymmetries should remain a component of testing batteries. Thus, future studies may need to evaluate the specificity of different dynamic balance assessments and sports performance.

### 4.4 Limitations

The current study had several limitations. First, nutrition, hydration, sleep, and training cycles were not controlled. Second, training status and injury history factors were not controlled in the current study. These factors could also contribute to performance as well. Second, the preferred legs and arms were defined as the preferred kicking leg or throwing arm. The preferred limb defined in the current study should not be considered the dominant limb or the better-performance limb in the assessment tasks. The close-chain jumping and pushing tasks may not involve the same preferred limbs as in the open-chain kicking and throwing motion (41). Furthermore, the number of participants for each age and sex group was relatively low, and future studies may need to recruit a larger number of participants to confirm the current study's outcome. The results of the current study should not be generalized to other populations and sports. Thus, future longitudinal and cohort studies may need to confirm the results from the current study.

## 5 Conclusions

As age increased, young soccer players in the United States demonstrated decreased landing forces, increased jump displacement from CMJ-AS, and increased normalized peak forces during push-ups in both males and females. Male and female players demonstrated similar changes as a function of age in most assessments. Bilateral asymmetries were small and non-significantly different across ages and sexes. Soccer players may benefit from incorporating more upper and lower extremity dynamic balance training to improve dynamic balance over time to enhance sports performance and decrease injury risk as they progress to higher levels of competition. Landing technique training may be implemented for younger soccer players to decrease the high impact landing forces and landing related injuries. Asymmetries and their relationships with injury risk should be evaluated on an individual basis, as their relationships with age and sex were weak. Future longitudinal studies to quantify the effects of age and sex and cohort studies to evaluate the relationships between strength and dynamic balance and injury risk in different age and sex groups are warranted.

## Data Availability

The de-identified raw data supporting the conclusions of this article will be made available by the authors. Requests to access the datasets should be directed to the corresponding author.
